# Risk Factors Associated With Rhegmatogenous Retinal Detachment

**DOI:** 10.7759/cureus.23201

**Published:** 2022-03-15

**Authors:** Arslan Shahid, Kashif Iqbal, Saad M Iqbal, Zia Ghaffar, Moneeb Tariq, Mohammad Jehanzeb Tahir, Fawad U Rahman, Usama Raheem, Jawad B Butt, Kiran Abbas

**Affiliations:** 1 Department of Ophthalmology, Layton Rehmatullah Benevolent Trust Free Eye Hospital, Lahore, PAK; 2 Department of Ophthalmology, Nawaz Sharif Medical College, Gujrat, PAK; 3 Department of Ophthalmology, Sargodha Medical College, Sargodha, PAK; 4 Department of Ophthalmology, Dera Ghazi Khan Medical College, Dera Ghazi Khan, PAK; 5 Department of Medicine, Jinnah Postgraduate Medical Centre, Karachi, PAK

**Keywords:** ophthalmology, intraocular surgery, trauma, lattice degeneration, rhegmatogenous retinal detachment

## Abstract

Background

Even though significant improvements have been made in the field of ophthalmology, retinal detachment is still an ever-increasing issue in both developing and developed countries. The present study evaluated the risk factors of rhegmatogenous retinal detachment (RRD).

Methodology

A cross-sectional study was conducted at a tertiary care center between June 2020 and March 2021. A total of 100 patients diagnosed with RRD were enrolled in the study. Patients with inconclusive diagnoses and multiple comorbidities were excluded from the study. A detailed history was taken, including previous surgery and ocular trauma or infections. A comprehensive ocular examination was conducted by an experienced ophthalmologist, including a dilated fundus examination. The causes and type of RRD were documented.

Results

In the study, a majority of the patients were males, with a mean age of 37.84 (18.29) years and a range of 5-74 years. The majority of those with total RRD were males, i.e., 37%; however, the difference was statistically insignificant (p=0.476). The study revealed that most of the RRD were diagnosed in patients <45 years of age; however, the difference was not statistically significant (p<0.227). The most frequent cause of RRD was lattice degeneration. While 23% of patients with RRD had a history of ocular trauma, uncomplicated phaco was detected in 17 cases. It was found that patients aged less than 45 years more frequently reported ocular trauma as the cause of RRD (p=0.004). Similarly, the cause of RRD was also significantly associated with the type of RRD (p=0.001).

Conclusion

The present study concludes that lattice degeneration, ocular trauma, uncomplicated, and complicated phaco are the main predisposing factors associated with RRD. Furthermore, the majority of the patients were males in their late thirties. Age, gender, and eye involvement were not significantly associated with the type of RRD.

## Introduction

One of the most common kinds of retinal detachment is rhegmatogenous retinal detachment (RDD) - caused by a tear in the retina that leads to the separation of the supporting retinal pigment epithelium (RPE) from the neurosensory retina (NSR). Despite significant advances in management, functional outcomes are still inadequate, with only 42% of individuals regaining 20/40 vision and just 28% if the macula is affected [1].

Even though the NSR and RPE do not have anatomical connections, mild mechanical forces (e.g., fluid pressures, intraocular, interphotoreceptor matrices, interdigitations between the microvilli and photoreceptors), and metabolic pressures (such as oxygenation) foster adhesions between these two layers [2]. A retinal detachment could develop when these adhesive forces are compromised. Exudative (serous), tractional, rhegmatogenous, and mixed tractional/rhegmatogenous retinal detachment are the four basic kinds of retinal detachment, each termed based on pathogenesis. RRD can emerge from atrophic perforations or retinal tears at various physiological and pathological regions of robust vitreoretinal attachment during posterior vitreous detachment (PVD) [3].

As age increases, PVD is more frequent as the vitreous undergoes substantial changes with age [4]. About 11% of 60 to 69-year-olds and 46% among 80 to 89 years old experience the disease [5]. The elderly population has the highest yearly incidence rate of RRD, and some evidence demonstrates a bimodal distribution and a transient surge at younger ages (20 to 30 years) due to severe myopia [5-7]. Among the age groups of 60 to 69 years, the incidence of RRD is the highest and ranges between 19 and 27 per 100,000 individuals [8].

Some statistics suggest a gender distribution similar to that of the general public [4,5]. The majority of studies show a predominance of males [8] (male to female ratio of 1.3:1 to 2.3:1), while a few indicate that women predominate in the phakic non-traumatic category (male to female ratio of 1:1.16 to 1:1.4) [9,10].

There is a scarcity of local literature on the subject. Therefore, the present study was conducted at a tertiary care center to explore the risk factors associated with RRD. The goal of the study was to evaluate the sociodemographic and clinical factors associated with RRD among the Pakistani population.

## Materials and methods

Study design

A cross-sectional study was performed after approval was procured from the institutional ethical committee (Reference # GEN-LRBT/865) prior to the start of the data collection. The study was conducted at the Layton Rahmatullah Benevolent Trust (LRBT) between June 2020 and May 2021.

Sampling technique

A non-probability convenience sampling technique was applied for the recruitment of the participants. For the sample size estimation, an online sample size calculator was used. By keeping the incidence of RRD at 20% in patients with a history of ocular trauma, a margin of error of 6.58%, a confidence level of 90%, and a sample size of 100 were obtained [11].

The study included all patients with RRD, irrespective of gender, aged between 5 and 74 years or more, and diagnosed by a specialist/consultant ophthalmologist. Patients with serous retinal or tractional detachment and RRD with vitreous leakage were excluded from the study.

Data collection procedure

Data collection was initiated by all the participants after obtaining informed verbal consent. All demographic characteristics, including identity, gender, and age, were documented as well. A detailed history was obtained from the patient or their attendants. Upon initial assessment, a previous record of cataract surgery was verified. A slit lamp and a dilated fundus examination were performed preoperatively to assess the type of retinal detachment and associated factors as mentioned above. All data were collected on a predesigned pro forma.

Data analysis

Statistical Package for the Social Sciences version 26 (SPSS, IBM Corp., Armonk, NY) was used for statistical analysis. For quantitative data such as age and frequency, mean and standard deviation were calculated. For categorical characteristics such as gender, eye involvement, lattice degeneration, injury, and intraocular surgery, percentages were computed. To determine the effects of modifiers on the mentioned characteristics, stratification was done using the chi-square test concerning eye involvement, age, and gender. A p-value of less than 0.05 was considered significant.

## Results

A total of 100 patients were enrolled in the study with a majority of the males. Mean age of 37.84 (18.29) years was noted with a range of 5-74 years. The mean age of patients did not vary significantly with respect to gender (p=0.797). The most common type of RD was the total RD with a frequency of 53 cases followed by inferior RD with 19 cases. The majority of those with total RRD were males, i.e., 37% however, the difference was statistically insignificant (p=0.476). The study revealed that most of the RRD were diagnosed in patients <45 years of age, however, the difference was not statistically significant (p=0.227).

The most frequent cause of RRD was lattice degeneration (36%) followed by a history of ocular trauma (23%). All causes are summarized in Table 1.

**Table 1:** Causes of rhegmatogenous retinal detachment

Causes	Frequency (%)
Complicated Phaco	11 (11%)
Extracapsular cataract extraction	2 (2%)
Iatrogenic	1 (1%)
Intracapsular cataract extraction	1 (1%)
Lattice degeneration	36 (36%)
Post-intravitreal injection	1 (1%)
Posterior vitreous detachment	1 (1%)
Refractive surgery	2 (2%)
Trauma	23 (23%)
Uncomplicated cataract	1 (1%)
Uncomplicated phaco	17 (17%)
YAG capsulotomy	4 (4%)

The four most frequent risk factors for RRD were lattice degeneration, ocular trauma, and uncomplicated or complicated phaco. Table 2 illustrates the relationship between risk factors for RRD and sociodemographic and clinical parameters. It was found that patients aged less than 45 years more frequently reported ocular trauma as the cause of RRD (p=0.004). Similarly, the cause of RRD was also significantly associated with the type of RRD (p=0.001).

**Table 2:** Risk factors for rhegmatogenous retinal detachment

Characteristics	Factors				p-Value
	Complicated phaco	Lattice	Trauma	Uncomplicated phaco	
Age					0.004
<45 years	3 (27.3%)	0 (0.0%)	21 (91.3%)	6 (35.3%)	
>45 years	8 (72.7%)	1 (100.0%)	2 (8.7%)	11 (64.7%)	
Gender					0.189
Male	8 (72.7%)	19 (52.8%)	19 (82.6%)	3 (75.0%)	
Female	3 (27.3%)	17 (47.2%)	4 (17.4%)	1 (25.0%)	
Eye causes					0.991
Left	4 (36.4%)	14 (38.9%)	8 (34.8%)	10 (58.8%)	
Right	7 (63.6%)	22 (61.1%)	15 (65.2%)	7 (41.2%)	
Diagnosis					0.001
Funnel retinal detachment	0 (0.0%)	0 (0.0%)	0 (0.0%)	0 (0.0%)	
Inferior retinal detachment	1 (9.1%)	9 (25.0%)	6 (26.1%)	3 (17.6%)	
Nasal retinal detachment	0 (0.0%)	1 (2.8%)	0 (0.0%)	0 (0.0%)	
Subtotal retinal detachment	0 (0.0%)	4 (11.1%)	2 (8.7%)	2 (11.8%)	
Superior retinal detachment + macular hole	0 (0.0%)	1 (2.8%)	0 (0.0%)	0 (0.0%)	
Superior retinal detachment	3 (27.3%)	2 (5.6%)	1 (4.3%)	1 (5.9%)	
Supratemporal retinal detachment	0 (0.0%)	1 (2.8%)	0 (0.0%)	0 (0.0%)	
Temporal retinal detachment	0 (0.0%)	1 (2.8%)	0 (0.0%)	2 (11.8%)	
Total retinal detachment	7 (63.6%)	17 (47.2%)	13 (56.5%)	9 (52.9%)	
Total retinal detachment + macular hole	0 (0.0%)	0 (0.0%)	1 (4.3%)	0 (0.0%)	

## Discussion

RRD impacts approximately 12 out of 100,000 individuals in the general public (0.01% yearly risk), with an absolute lifetime risk of 0.6% (up to 60 years old) [10,11]. Cataract surgery, significant myopia, severe optical trauma, ophthalmic infections, lattice degeneration, and glaucoma are only a few of the well-known key predictors that have been related to the development of RRD [11-14].

Our study findings showed more male patients than females with a mean age of 37.84 (18.29) years, with a range of 5-74 years. According to other reports, 3.1% of RRD patients had a history of trauma, specifically RRD patients in Ethiopia. In South Africa, the proportion was 30%, while in Kenya, it was 8% [15]. In our study, the most common cause of RRD was lattice degeneration, with a frequency of 36%. This was consistent with published literature. For instance, according to research conducted in Japan, a greater percentage of lattice degeneration, i.e., 60-65% was revealed [10].

We further found that the majority of the cases of RRD were diagnosed in patients younger than 45 years of age. This finding was in contrast to a recent local study which revealed that most of the RRD cases were found in patients aged approximately 50 years [16]. As mentioned earlier, this study found 36% of the population with RRD had lattice degeneration. Current evidence strongly reports that up to 30% of RRD patients have lattice degeneration [17]. However, this finding is inconsistently reported in the literature. According to a study conducted in the United Kingdom, the overall prevalence of lattice degeneration was much higher, i.e., 45.7 ± 20.3%, while the prevalence of myopia was 47.28 ± 12.59% [18].

In the context of the subcontinent, the studies reported the rate of macular detachment to be around 90% [19]. The inferior half was discovered to be more impacted than the superior half, while the temporal half was found to be more affected than the nasal half [20]. In some cases of RRD, the detachment is not identified due to several factors. The small size of the break, peripherally localized breaks, limited vision on indirect ophthalmoscopy owing to intraocular lens aberrations, and obscuration by peripheral capsular opacification are all factors involved in the inability to identify primary breaks in aphakic/pseudophakic eyes [21]. 

The study also countered several limitations during the study. First, the limited sample size of the study posed a great challenge for the authors. Therefore, since it was a single-center study, the current findings cannot be generalized to a larger population. Large-scale and multi-center studies are warranted to explore the predisposing factors in a more comprehensive manner.

## Conclusions

The present study concludes that lattice degeneration, ocular trauma, uncomplicated, and complicated phaco are the main predisposing factors associated with RRD. Furthermore, the majority of the patients were males in their late thirties. Age, gender, and involvement of the eye were not significantly associated with the type of RRD.
